# Headgear mandates in high school girls’ lacrosse: investigating differences in impact rates and game play behaviors

**DOI:** 10.1080/07853890.2024.2362862

**Published:** 2024-06-20

**Authors:** Shane V. Caswell, Patricia M. Kelshaw, Samantha L. Hacherl, Andrew E. Lincoln, Daniel C. Herman

**Affiliations:** a School of Kinesiology, Athletic Training Program, George Mason University, Manassas, VA, USA; b Sports Medicine Assessment, Research & Testing Laboratory, George Mason University, Manassas, VA, USA; c Virginia Concussion Intiative, George Mason University, Manassas, VA, USA; d Department of Kinesiology, Brain Research & Assessment Initiative of New Hampshire, University of New Hampshire, Durham, NH; e Special Olympics International, Washington, DC; f Department of Physical Medicine and Rehabilitation, University of California at Davis, Sacramento, CA, US

**Keywords:** Concussion, helmets, risk-compensation, policy, females

## Abstract

**Background/objective:**

Headgear designed to protect girls’ lacrosse athletes is widely available and permitted for voluntary use; however, it remains unknown how policies mandating headgear use may change the sport and, particularly regarding impacts during game-play. Therefore, this study compares the impact rates and game play characteristics of girls’ high school lacrosse in Florida which mandates headgear use (HM), with states having no headgear mandate (NHM).

**Materials and methods:**

Video from 189 randomly-selected games (HM: 64, NHM: 125) were analyzed. Descriptive statistics, Impact Rates (IR), Impact Rate Ratios (IRR), Impact Proportion Ratios (IPR), and 95% Confidence Intervals (CI) were calculated. IRRs and IPRs with corresponding CIs that excluded 1.00 were deemed statistically significant.

**Results:**

16,340 impacts (HM:5,821 NHM: 10,519; 86.6 impacts/game, CI: 88.6–93.3) were identified using the Lacrosse Incident Analysis Instrument (LIAI). Most impacts directly struck the body (*n* = 16,010, 98%). A minority of impacts directly struck a player’s head (*n* = 330, 2%). The rate of head impacts was significantly higher in the HM cohort than NHM cohort (IRR = 2.1; 95% CI = 1.7–2.6). Most head impacts (*n* = 271, 82%) were caused by stick contact in both groups. There was no difference in the proportion of penalties administered for head impacts caused by stick contact between the HM and NHM cohorts (IPR IRRHM/NHM = 0.98; CI = 0.79–1.16). However, there was a significantly greater proportion of head impacts caused by player contact that resulted in a penalty administered in the HM cohort (IPR = 1.44 CI = 1.17–1.54)

**Conclusion:**

These findings demonstrate that mandating headgear use was associated with a two-fold greater likelihood of sustaining a head impact during game play compared to NHM states. A majority of head impacts in both HM and NHM states were caused by illegal stick contact that did not result in penalty.

## KEY MESSAGES

- High school girls’ lacrosse athletes participating in a state with a headgear mandate was twice as likely to sustain a head impact than those participating in states without headgear mandates.
- Stick contact remains the most common mechanism of head impacts in girls’ lacrosse, regardless of mandating headgear.
- Regardless of whether headgear was or was not mandated, most head impacts caused by stick contact did not result in a penalty.

## Introduction

Lacrosse is among the fastest growing sports among girls in the United States (US) and globally [1–3]. Although a non-contact sport, girls’ lacrosse game play can expose participants to head impacts [4–6]. Head and face injuries are the most common game-related injuries in high school girls’ lacrosse, accounting for 0.92 injuries per 1000 athlete exposures (AE). Concussions account for a majority of these injuries (0.83 injuries per 1000 AEs), with stick-to-head contact being the leading cause of concussions in high school girls’ lacrosse [7]. Efforts to reduce the risk of head injury are not new to lacrosse. In 1986, concerns about head injuries led the Massachusetts Interscholastic Athletic Association to mandate that ice hockey helmets be worn by all girls’ high school lacrosse athletes. The mandate was rescinded 10 years later, based on anecdotal evidence suggesting that helmets were associated with risk compensation and increased aggressive game play behaviors that elevated injury risk [8,9]. Since this time considerable research has contributed to an improved understanding of head and facial injuries in girl’s lacrosse [5, 7, 10–12]. In 2017, headgear designed to address incidental stick and ball to headgear impacts in accordance with the ASTM International F3137 performance standard became commercially available [13,14]. Nevertheless, considerable debate persists about the benefits and consequences of mandating headgear in girl’s lacrosse [8,9, 15–17]. At present, headgear are considered optional equipment for girls’ high school lacrosse in the US [18,19] with the exception of Florida where headgear meeting ASTM International F3137 are mandated [20–22]. As such, there has been a growing need to better understand the effect of headgear use on head impacts and the nature of game-play in the sport.

There is a need for empirical data evaluating the effects of headgear use on game play behaviors and player safety. However, little research has been conducted. A small study suggested that headgear use may not be associated with changes in game play behaviors or the rates of head impacts [4]. Caswell et al. [4] conducted a single cohort interventional study of headgear use with a convenience sample of a single high school girls’ lacrosse team, and observed that while the impact frequencies did not change, the magnitudes of impacts were slightly lower during the intervention of headgear. However, the wearable accelerometers used in this study to quantify head impact magnitudes are vulnerable to considerable measurement error [6], thus limiting the validity of the findings, particularly given the small sample size. In support of these findings, two recent epidemiological studies suggest that headgear mandates may be associated with lower rates of concussion [23,24]. However, other studies have demonstrated that some stakeholders in lacrosse perceive headgear as being responsible for negative effects on game play and player safety [25–27]. According to the Peltzman Effect, mandating headgear in girls’ lacrosse may encourage players to engage in riskier or more aggressive game behaviors because they feel protected, which could explain these perceived negative effects [28]. Collectively, the findings from the limited available research do not provide enough information for policy makers to evaluate headgear mandates.

To better understand the effects of required headgear use in girls’ high school lacrosse, it is important to describe the frequency of head and body impacts, as well as gameplay behaviors in both mandated and non-mandated conditions. Video-analysis observational studies have been used in numerous sport settings to identify mechanisms and measure head impacts and describe game play behaviors [29–33]. Additionally, video-analysis allows for larger and more geographically representative studies that provide an effective approach to measuring head impact exposure and evaluating potential changes in game play behaviors after interventions [32]. Therefore, the purpose of this study was to use video-analysis to evaluate if differences in the rates of head and body impacts and game play behaviors exist between high school girls’ lacrosse players participating in Florida which mandates headgear use and other states that do not mandate headgear use. We hypothesized that there would be no differences in the rates of head and body impacts, their mechanisms, and associated penalties administered during games played in Florida and between other states without a headgear mandate.

## Methods

### Sample

This cohort study compared the rate of impacts and game play characteristics of girls’ high school varsity lacrosse (GLAX) in Florida, which is the only US state athletic association with rules mandating headgear use (Headgear Mandate, herein ‘HM’) with other US states which have no rules mandating headgear use (No Headgear Mandate, herein ‘NHM’) [18,20]. GLAX game videos were obtained from the NFHS Network (Play On Sports, Atlanta GA). The NFHS Network is a subscription-based service that offers on-demand videos of 27 high school sports from more than 10,000 high schools nationwide [34]. The research team established a comprehensive sampling frame by using a list provided by the NFHS Network, containing all GLAX games recorded from participating high schools (grades 9–12) during the 2020 season that preceded this study. In total, the list was comprised of 8848 GLAX games (HM = 6%; *n* = 554, NHM = 94%; *n* = 8294) from 32 states. This frame was used to conduct a stratified random sampling procedure that proportionally selected GLAX games from both the HM and NHM cohorts that were played during the 2021 and 2022 lacrosse seasons. It is worth noting that only varsity-level GLAX games were considered, as a limited number of junior varsity games were available on the NFHS Network during the study. Within the HM and NHM cohorts, varsity GLAX games available on the NFHS Network were randomly selected with a probability proportional to the total number of games from schools located in different geographic locations and different timepoints of the competitive season. Approximately 1:2 ratio of HM:NHM games. The final sample consisted of video of *N* = 189 varsity GLAX games (HM: *n* = 64, 34%; NHM: *n* = 125, 66%) (See Supplemental Table). Prior to video analysis, games were reviewed for video quality. Games with poor video quality were removed and another game was randomly selected. Ethical approval for the study methods including a waiver of participant consent was approved by the George Mason University Institutional Review Board [500707-17].

### Video analysis

We used a modified version of the Lacrosse Incident Analysis Instrument (LIAI) and methodology for reviewing game video and identifying impact events for analysis as previously described [5, 35]. Our approach reviewed video footage for both the home and visiting teams for each entire game. All observed impacts were coded to identify player position (midfield, defense, attack), impact mechanism (player, stick, ball, ground), body location (head, body), and whether a penalty was administered. All raters classified an average of 86% agreement in alignment with prior recommendations [31].

### Statistical analysis

All data was analyzed using R software (version 4.0.2, R Foundation for Statistical Computing). Descriptive statistics (counts and proportions) and impact rates (IR) with 95% confidence intervals (CI) were calculated for impacts observed. The IRs were calculated as the total number of impacts divided by the total number of games for the for the HM and NHM cohorts, respectively. The formula was as follows:

$$\text{Impact Rate }(\text{IR})=\frac{\text{Σ Impacts}}{\text{Σ Games}}$$

To evaluate potential differences in IR associated with mandated headgear use, impact rate ratios (IRRs) were computed to compare the IR between the HM and NHM cohorts by game play characteristics of player position and impact location and mechanism. The formula for calculating the IRR was as follows:

Impact  Rate  Ratio=Σ HM  impactsΣ HM  gamesΣ NHM  impactsΣ NHM  games

Stick and player contact is illegal in girls’ lacrosse; therefore we computed impact proportion ratios (IPRs) for all player and stick impacts in which a penalty was or was not administered for both the HM and NHM cohorts. The following is an example of an IPR comparing the proportion of penalties called for head impacts caused by stick in HM games versus NHM games:

Impact  Proportion  Ratio=Σ HM  stick  impacts  to  the  head Σ HM  gamesΣ NHM  stick  impacts  to  the  head Σ NHM  games

All IRRs and IPRs with 95% CI that excluded 1.00 were considered statistically significant. IRRs and IPRs were not calculated when groups had less than 10 impacts to avoid inaccurate effect estimates.

## Results

### Headgear mandate comparison

The rate of head impacts was significantly higher in the HM than the NHM cohort (HM IR: 2.7 vs. NHM IR: 1.3 impacts/game; IRR_HM/NHM_: 2.1, CI: 1.7–2.6) (Table 1). Within position midfielders (HM IR: 1.6 vs. NHM IR: 0.8 impacts/game; IRR_HM/NHM_: 2.0, CI: 1.5–2.7) and attackers (HM IR: 0.9 vs. NHM IR: 0.3 impacts/game; IRR_HM/NHM_: 3.0, CI: 2.0–4.5) in HM cohort had significantly higher rates of head impacts than their NHM counterparts. See Table 2 for additional descriptive information regarding impacts by player position within the HM and NHM cohorts. Most head impacts (82%) were caused by stick (HM: *n* = 145, 84.3%; NHM: *n* = 126, 79.7%) followed by player (12%) contact (HM: *n* = 20, 11.6%; NHM: *n* = 19, 12.0%). The rates of head impacts caused by either stick or player contacts were significantly higher in the HM than the NHM cohort (Stick IR: 2.3 vs 1.0; IRR_HM/NHM_: 2.2, CI:1.8–2.9; Player IR: 0.3 vs 0.2; IRR_HM/NHM_: 2.1, CI: 1.1–3.9) (Table 3).

**Table 1.** Head, body and overall impacts in girls’ high school lacrosse.

	Headgear mandate			No headgear mandate		
	Head	Body	Overall	Head	Body	Overall
Games	64	64	64	125	125	125
Impact count	172	5649	5821	158	10361	10519
IR (95% CI)	2.7 (2.3–3.1)	88.3 (86.0–91.0)	90.6 (88.6–93.3)	1.3 (1.0–1.5)	82.9 (81.3–84.5)	84.2 (82.6–85.8)
IRR (95% CI)	2.1 (1.7–2.6)*	1.1 (1.0–1.1)	1.1 (1.0–1.1)			

Note: IR = impact rate per game; IRR = impact rate ratio (HM Cohort is reference), *statistically significant.

**Table 2.** Head and body impacts by headgear cohort and player position.

	Headgear mandate (HM)		No headgear mandate (NHM)		HM vs NHM
Position	n (%)	IR (95% CI)	n (%)	IR (95% CI)	IRR (95% CI)
Head impacts					
Attacker	58 (33.7)	0.9 (0.7–1.2)	38 (24.1)	0.3 (0.2–0.4)	3.0 (2.0–4.5)*
Midfield	105 (61.0)	1.6 (1.3–2.0)	101 (63.9)	0.8 (0.7–1.0)	2.0 (1.5–2.7)*
Defender	9 (5.2)	0.1 (0.1–0.3)	19 (12.0)	0.2 (0.1–0.2)	0.9 (0.4–2.0)
Body impacts					
Attacker	1245 (22.0)	19.5 (18.4–20.6)	2,120 (20.5)	17.0 (16.2–17.7)	1.1 (1.1–1.2)
Midfield	3612 (63.9)	56.4 (54.6–58.3)	6667 (64.3)	53.3 (52.1–54.6)	1.1 (1.0–1.1)
Defender	792 (14.0)	12.4 (11.5–13.3)	1,574 (15.2)	12.6 (12.0–13.2)	1.0 (0.9–1.1)

Note: IR = impact rate per game; IRR = impact rate ratio (HM Cohort is reference), *statistically significant.

**Table 3.** Impacts to the head and body by headgear cohort and mechanism.

	Headgear mandate (HM)		No headgear mandate (NHM)		HM vs NHM
Mechanism	n (%)	IR (95% CI)	n (%)	IR (95% CI)	IRR (95% CI)
Head impacts					
Ball	3 (1.7)	0.0 (0.0–0.1)	4 (2.5)	0.0 (0.0–0.1)	
Stick	145 (84.3)	2.3 (1.9–2.7)	126 (79.7)	1.0 (0.8–1.2)	2.2 (1.8–2.9)*
Player	20 (11.6)	0.3 (0.2–0.5)	19 (12.0)	0.2 (0.1–0.2)	2.1 (1.1–3.9)*
Ground	4 (2.3)	0.1 (0.0–0.1)	9 (5.7)	0.1 (0.0–0.1)	
Body impacts					
Ball	11 (0.2)	0.2(0.1–0.3)	14 (0.1)	0.1 (0.1–0.2)	1.5 (0.7–3.4)
Stick	2034 (36.0)	31.8 (30.4–33.2)	3740 (36.1)	29.9 (29.0–30.9)	1.1 (1.0–1.1)
Player	2911 (51.5)	45.5 (43.9–47.2)	5359 (51.7)	42.9 (41.7–44.0)	1.1 (1.0–1.1)
Ground	692 (12.2)	10.8 (10.0–11.6)	1246 (12.0)	10.0 (9.4–10.5)	1.1 (0.1–1.2)
Other	1 (0.0)	0.0 (0.0–0.1)	2 (0.0)	0.0 (0.0–0.1)	

Note: IR = impact rate per game; IRR = impact rate ratio (HM Cohort is reference), *statistically significant.

A majority (61%) of head impacts caused by stick or player contact did not result penalty for both cohorts (HM: *n* = 100, 75%; NHM: *n* = 88, 79%). There was no difference in the proportion penalties administered for head impacts caused by stick contact between the HM and NHM cohorts (IPR_HM/NHM_: 1.0, CI:0.8–1.2). However, there was a significantly greater proportion of head impacts caused by player contact that resulted in a penalty administered in the HM than the NHM cohort (IPR: 1.4, CI: 1.2–1.5) (Table 4). The rate of impacts sustained to the body did not vary significantly between the HM and NHM cohorts (IR: 88.3 vs 82.9 respectively; IRR: 1.1, CI: 1.0–1.1). There were no differences in the rates of impacts sustained to the body within positions between the NM and NHM cohorts. Table 2 provides additional descriptive information regarding body impacts sustained by player position for the HM and NHM cohorts. Common mechanisms for body impacts were player (HM: *n* = 2911, 52%; NHM: *n* = 5359, 52%), followed by stick (HM: *n* = 2034, 36%; NHM: *n* = 3740, 36.1%) and then ground contacts (HM: *n* = 692, 12.2%; NHM: *n* = 1246, 12%). The rates of body impacts did not vary by mechanism between the HM and NHM cohorts (Table 3). A majority (81%) of body impacts caused by stick or player contact did not result penalty for both cohorts (HM: *n* = 3939, 80%; NHM: *n* = 7375, 81%). There were no significant differences in the proportions of penalties administered for impacts between HM and NHM cohorts associated with stick to body (IPR: 1.0, CI: 0.6–1.4) or player to body (IPR: 0.1, CI: 0.6–1.4) contacts.

**Table 4.** Penalties administered for stick and player impacts to the head and body by headgear mandate cohort.

		Headgear mandate (HM)		No headgear mandate (NHM)		HM vs NHM
	Mechanism	n (%)	IR (95% CI)	n (%)	IR (95% CI)	IPR (95% CI)
Penalty	Head					
	Stick	60 (41.4%)	0.9 (0.7–1.2)	53 (31.4%)	0.3 (0.2–0.4)	0.1 (0.8–1.2)
	Player	5 (25.0%)	0.1 (0.1–0.3)	3 (27.3%)	0.2 (0.1–0.2)	1.4 (1.2–1.5)
	Total	65 (39.4%)	1.6 (1.3–2.0)	56 (31.6%)	0.8 (0.7–1.0)	
	Body					
	Stick	617 (23.1%)	9.6 (8.9–10.4)	1040 (19.4%)	8.3 (7.8–8.8)	1.0 (0.6–1.4)
	Player	389 (19.1%)	6.1 (5.5–6.7)	684 (18.3%)	5.5 (5.1–5.9)	0.1 (0.6–1.4)
	Total	1006 (20.1%)	15.7 (14.8–16.7)	1724 (19.1%)	13.8 (13.2–14.1)	
No Penalty	Head					
	Stick	85 (58.6%)	19.5 (18.4–20.6)	113 (68.6%)	17.0 (16.2–17.7)	0.9 (0.7–1.1)
	Player	15 (75.0%)	12.4 (11.5–13.3)	8 (72.7%)	12.6 (12.0–13.2)	2.3 (2.1–2.5)*
	Total	100 (60.6%)	56.4 (54.6–58.3)	121 (68.4%)	53.3 (52.1–54.6)	
	Body					
	Stick	2294 (76.9%)	35.8 (34.4–37.3)	4319 (80.6%)	34.5 (33.6–35.6)	1.0 (0.6–1.4)
	Player	1645 (80.9%)	25.7 (23.5–27.0)	3056 (81.7%)	24.4 (23.6–25.3)	1.0 (0.6–1.4)
	Total	3939 (79.9%)	61.5 (59.6–63.5)	7375 (80.9%)	59.0 (57.7–60.4)	

Note: IR = impact rate per game; IPR = impact proportion ratio (HM cohort is reference), *statistically significant.

### Discussion

We used game video to compare head impact rates and game play behaviors of high school girls’ varsity lacrosse players participating in Florida, where headgear is mandated, with those participating in other states where headgear is not mandatory. Our findings revealed that girls who played high school varsity lacrosse in Florida, had twice the incidence of head impacts caused by stick or player contact during games, compared to those playing in states without a headgear mandate. We also observed that over half (61%) of all head impacts in both the HM and NHM cohorts did not result in a penalty. However, we observed no other significant differences in the rates of head or body impacts, mechanisms, or if a penalty was administered. Collectively, these findings suggest that our hypothesis, which predicted no differences in the rates of head or body impacts, their mechanisms, and associated penalties between states with and without a headgear mandate, is partially rejected.

### Headgear mandate and head impacts

Numerous prior studies [7, 11,12, 36–41] have shown concussions to be a common game-related injury in high school girl’s lacrosse, with most resulting from head impacts caused by a stick, a ball, or another player. Since 2017, female high school lacrosse players have been allowed to voluntarily wear headgear that meets the ASTM International F3137 standard, designed to address incidental stick and ball impacts [13,14]. However, a limited number of laboratory [42,43] and epidemiological [23,24] research studies have evaluated the effectiveness of lacrosse headgear in mitigating impact severity and reducing the incidence of concussion. To date, only a single published research study has evaluated the effects of headgear use on impacts during high school girls’ lacrosse game play. Caswell et al. [4] prospectively studied a single varsity girls’ high school lacrosse team before and after the adoption of headgear. They found no difference in the rates of head or body impacts after adopting headgear. However, their study had some limitations, as it focused on a small sample of voluntarily early headgear adopters who competed against other teams that did not wear headgear. In contrast, our study leveraged a nationally representative dataset of high school girls’ lacrosse game videos in which both teams were either required to wear or not wear headgear as mandated by their respective state athletic association rules. We found that athletes who played in Florida, where the use of headgear is mandatory for all players, experienced significantly more head impacts compared to those playing in other states that did not have such a mandate. Moreover, we noted that the positions of midfield and attack accounted for the significantly higher rate of head impacts observed in the HM cohort.

With regard to mechanisms of impacts, Caswell et al. [4] found no differences associated with head impact mechanisms before or after the implementation of headgear. In contrast, we found the rates of both stick-to-head (IRR: 2.3 HM vs. 1.0) and player-to-head (IRR: 0.3 HM vs. 0.2) impacts per game in HM cohort to be more than twice that of NHM cohort. This finding may support opponents of headgear who contend that mandating headgear use will result in more aggressive game-play and increase head impacts. However, we suggest caution as the proportion of head impacts was small (2% of all impacts) as were the overall rates for both the HM (2.7 head impacts per game) and NHM cohorts (1.3 head impacts per game). Consequently, the observed statistical significance in player-to-head impacts between HM and NHM cohorts may not necessarily translate into meaningful practical implications from a game-play perspective. Additionally, we observed no other significant differences between impact rates or mechanisms between HM and NHM cohorts.

Interestingly, our findings indicated that only 2% of all head impacts in girls’ lacrosse were caused by a ball-to-head mechanism. Previous studies that examined injury rates in girls’ lacrosse have categorized injury mechanisms as equipment-related, without separating ball and stick injury mechanisms [41, 44]. This has resulted in a lack of understanding about the relative risks associated with these two distinct types of equipment impacts. If concussions resulting from ball-to-head impacts are more common than our impact data suggest, it could guide future investigations evaluating headgear effectiveness in reducing the risk of concussions caused by these different mechanisms.

### Headgear mandate and game play

Girls’ lacrosse rules permit only stick-to-stick contact and minimal body contact, making it an incidental contact sport. Considerable debate exists about mandating headgear in girls’ lacrosse, citing potential changes in gameplay associated with its use may increase the risk of injury. Conversely, advocates believe that mandating headgear will lower the risk of head and facial injuries including concussions [13, 15]. Caswell et al. [4] compared of the distribution of impact mechanisms before and after the adoption of headgear. They observed no differences in the rates of impacts by mechanism or the proportion of impacts that resulted in a penalty. Similarly, we found no difference in the proportion penalties administered for head or body impacts caused by stick contact between the HM and NHM cohorts.

We did observe that a greater proportion of head impacts caused by player contact resulted in penalties in the HM cohort. This may be indicative of more aggressive game play behaviors that contribute to illegal player-to-player head contacts in the HM cohort. However, the number of player-to-player head contacts was quite low in both the HM and NHM cohorts. Additionally, the absence of any significant differences in body impact rates and the proportion of penalties awarded for body impacts, between the HM and NHM cohorts complicates the interpretation of our findings. If mandating headgear for girls’ lacrosse players results in riskier and more aggressive game behaviors, as suggested by the Peltzman Effect [28], it is reasonable to expect that these changes would similarly affect the rates and proportions of penalties administered for both head and body impacts. However, it is possible that the perception that headgear provides increased protection may only apply to the head, leading players to engage in riskier and more aggressive game behaviors that involve their head more than other parts of their body.

Of more general concern is the low rate of penalties administered in both HM and NHM cohorts. Although stick and player contact are illegal in the sport, multiple prior studies have reported that stick and player impacts frequently do not result in penalty [4,5, 11]. We similarly observed that only 1 in 5 impacts to the head and body caused by stick or player contact resulted in a penalty. Collectively, these findings suggest that mandating headgear may not be a singularly effective intervention in reducing head impacts. Further investigation evaluating the efficacy of officials in identifying and penalizing prohibited stick and player contacts is needed. Officials play a pivotal role in modifying game-play behaviors through the administration of penalties [27]. It is therefore imperative to address this issue comprehensively and assess the feasibility of implementing penalties that effectively deter game play mechanisms most closely associated with head impacts and concussions, such as stick and player contact. Future research should prioritize the exploration of novel strategies (e.g. educational interventions, improved training acquisition, or increasing the density of on and/or off field officials) to enhance the enforceability of penalties in girls’ lacrosse. Such efforts could not only contribute to reducing the incidence of impacts and concussions, but also uphold the integrity of the game while safeguarding the well-being of its participants.

We found that girls who play lacrosse in Florida and are required to wear headgear were twice as likely to experience stick- and player-to-head impacts during games as compared to those who play in states without such mandates. This is a cause for concern, as these are common mechanisms of concussion in girls’ high school lacrosse. However, a nationwide study by Herman et al. [24] recently found that girls playing lacrosse in states without a headgear mandate had a 74% higher incidence of concussion during games than those playing in Florida. This suggests that mandating headgear use in girl’s high school lacrosse may be a protective factor that outweighs the increased rate of game-related head impacts to which athletes may be exposed. Further investigation into the mandates regarding headgear and the enforceability of penalties could provide valuable insights for individualized interventions. These findings could enable institutions to make informed decisions, potentially opting for either the enforcement of headgear mandates or enhancements in penalty enforceability, aligning with their available resources and the specific needs of their respective teams and/or states.

### Limitations

This study used a random sample of high school lacrosse game video from a nation-wide video-based streaming platform. While the video quality was evaluated prior to inclusion in the study, it is possible that video quality varied across different high schools. As video data used in this study was retrospectively analyzed, the researchers were not able to confirm standardization of collection procedures beyond those details provided by the NFHS Network. While our procedures aligned with prior research and we attained acceptable inter-rater reliability, the use of different raters introduces some degree of measurement error relating to impact counts and characterizing game play that are beyond our control. Additionally, although we randomly selected game videos from HM and NHM cohorts our findings may not be generalizable to all high school girls’ lacrosse game play. Lastly, a strength of our study design was that in using a video-based approach we could assess a larger and more representative national sample of game video while also permitting a more complete characterization of the entirety of game play by including both teams.

## Conclusions

In conclusion, our findings suggest that girls participating in high school varsity lacrosse in Florida had significantly higher rate of head impacts during games, particularly stick-to-head impacts, than those playing in states without a headgear mandate. Additionally, the rate of penalties assessed for illegal contact was low in both HM and NHM cohorts. These findings, along with previous research, highlight the need for further investigation into the recognition and enforcement of penalties for illegal head and body impacts, as officials are challenged to effectively recognize and respond to such violations. Future research and collaboration among governing stakeholders should prioritize exploring and implementing strategies to improve the enforcement of penalties in girls’ lacrosse.

## Supplementary Material

Supplemental Material

## Data Availability

The authors agree to make data supporting the results presented in their paper available upon reasonable request.
