# Supplementary material for: Headgear mandates in high school girls’ lacrosse: investigating differences in impact rates and game play behaviors
Source: Ann Med. 2024 Jun 20;56(1):2362862. doi: 10.1080/07853890.2024.2362862 (PMC11195452; doi:10.1080/07853890.2024.2362862)
Supplement: Supplemental Material [file IANN_A_2362862_SM3033.docx]

Supplemental Table. Counts of Game Video Included in Sample by State

| State | Total |
| --- | --- |
| Arizona | 2 |
| California | 7 |
| Colorado | 3 |
| Connecticut | 6 |
| Delaware | 5 |
| Florida* | 64 |
| Georgia | 2 |
| Idaho | 2 |
| Illinois | 3 |
| Kentucky | 2 |
| Maine | 2 |
| Maryland | 4 |
| Massachusetts | 1 |
| Michigan | 5 |
| Minnesota | 4 |
| New Hampshire | 3 |
| New Jersey | 7 |
| New Mexico | 1 |
| New York | 8 |
| North Carolina | 7 |
| Ohio | 5 |
| Oregon | 2 |
| Pennsylvania | 5 |
| Rhode Island | 7 |
| Tennessee | 3 |
| Texas | 4 |
| Utah | 2 |
| Vermont | 5 |
| Virginia | 7 |
| Washington | 5 |
| West Virginia | 3 |
| Wisconsin | 3 |
| **TOTAL** | **189** |

*HM Reference State
